# Ethnopharmacological survey of home remedies used for treatment of hair and scalp and their methods of preparation in the West Bank-Palestine

**DOI:** 10.1186/s12906-017-1858-1

**Published:** 2017-07-05

**Authors:** Abdel Naser Zaid, Nidal Amin Jaradat, Ahmad Mustafa Eid, Hamzeh Al Zabadi, Abdulsalam Alkaiyat, Saja Adam Darwish

**Affiliations:** 1 Department of Pharmacy, Faculty of Medicine and Health Sciences, An-Najah National University, P.O. Box 7, Nablus, Palestine; 2 Public Health Division, Faculty of Medicine and Health Sciences, An-Najah National University, P.O. Box 7, Nablus, Palestine

**Keywords:** Ethnopharmacology, Cosmetics, Cosmeceutical, Herbals, Minerals, Animals

## Abstract

**Background:**

Natural products have many uses and purposes, including those linked to pharmaceutics and cosmetics. The aim of this study was to investigate the use of natural remedies for the treatment of hair and scalp disorders in the West Bank, Palestine.

**Methods:**

An ethnopharmacological survey of herbal remedies and other natural products used in cosmetics and cosmeceuticals was carried out in the West Bank, Palestine. A questionnaire was distributed to 267 herbalists, traditional healers, hairdressers and rural dwellers. Collected information included: the names of plants and other natural products, the parts used, hair conditions, diseases and problems for which these products were used and also their methods of preparation. To identify the most important species used, the factor of informant’s consensus (F_ic_), fidelity level (Fl) and the use-value (UV) were calculated.

**Results:**

Collected data showed that 41 plants are utilized for the treatment of hair and scalp disorders, belonging to 27 families; among them Lamiaceae and Rosaceae, which were the most commonly used. Plant oils and their fruits are the most commonly used parts. Hair loss, dandruff, split hair endings and lice treatment, are reported as the most treated disorders. The number of plant species used consisted of 19, 14, 13, and again 13 with a factor of informant’s consensus (Fic) for these disorders corresponding to 0.93, 0.94, 0.95 and 0.92, respectively. Fl was 100% for many plants; the highest UV value (0.84) was registered for *Lawsonia inermis,* which belongs to the Lythraceae family.

**Conclusions:**

This study showed that many natural remedies are still used in Palestine for the treatment of scalp and hair disorders as well as for cosmeceutical purposes. This study is of great importance as it allows us to have a greater perspective on our folkloric use of these natural products. A combined scientific effort between informants and the scientific community, working in this field, may help in the discovery of new cosmetics, cosmeceutical and nutraceutical products.

## Background

Palestine, also known as the Holy Land, has great ethnic variability: Muslims, Christians, Druze, Jews from East and West and Samaritans. Such a variety has enriched its culture, especially that of a folkloric nature, herbal foods, medicines and cosmetics. Thus the holy land is a unique area in its ecological diversity due to its geographical location in the Mediterranean region. Varied zoogeographic, climatic, and phytogeographic zones cover Palestine hence generating great biological multi-diversity [1]. In addition to this, it has been an important international trade cross road since the ancient times, between the Eastern and Western worlds, further enriching its culture in herbal remedies and home uses [2–4].

Cosmetics such as perfumes and soaps have been used and developed by people for decades [5–8]. Based on the European Commission [9], cosmetic products have been defined, “as any substance or preparation intended to be placed in contact with the external parts of the human body or with the teeth and the mucous membranes of the oral cavity with a view exclusively or mainly to cleaning them, perfuming them, changing their appearance, and/or correcting body odors and/or protecting or keeping them in good condition”. Accordingly, cosmetics can be classified according to the following classes: (i) skin care cosmetics, (ii) makeup cosmetics, (iii) perfume and eau de cologne, (iv) hair care products, and (v) special-purpose cosmetics. The merging of pharmaceutics and cosmetics is known as cosmeceuticals, which consists of products with medicinal properties that shows beneficial topical actions and provides protection against degenerative skin conditions [10, 11].

Herbs, animals, and minerals provide a continuous source of food, medicines and cosmetics for humans, which have been long used in several forms including: (i) decoctions, (ii) syrups, (iii) liniments, (iv) powders, (v) infusions, (vi) gels and magmas, (vii) creams, pastes and ointments [12, 13]. In 1960, proof of medicinal herbal and plant use, in the Mediterranean area, was discovered, to be existing, in a cave from, approximately, 60,000 years ago [14]. More recently, people in both developed and developing countries utilize medicines and cosmetic preparations obtained from natural sources for the improvement of their health and aesthetic appearance [15, 16]. According to the World Health Organization (WHO), about 80% of populations in developing countries have utilized ethnomedicines for their health care. The plants, commonly used in domestic medicine and home remedies, by those of different cultures, are traditionally used for the remedy of hair and scalp diseases where the main focus, currently, is on their “cosmeceutical” purposes. However we need to appreciate that medical anthropological aspects of the etiology of some hair and scalp afflictions are very complex and are not always completely understood [17, 18].

## Methods

**Fig. 1:**
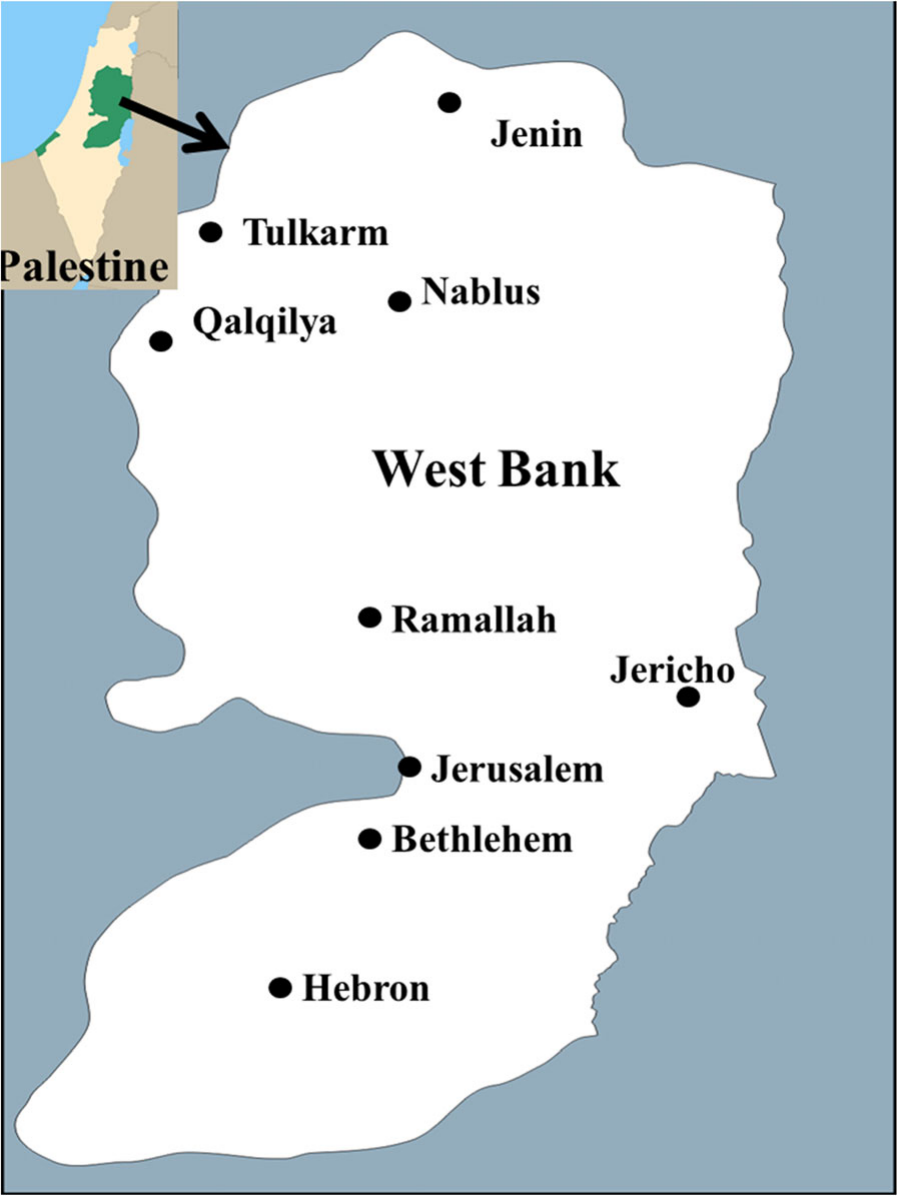
Map of the West Bank/ Palestine showing all surveyed regions

An ethnopharmacological survey on herbal, mineral and animal products, used as cosmetics or cosmeceuticals, in the treatment of hair and scalp disorders, was conducted from April to June 2016. The visited and interviewed areas consisted of all of the regions of the West Bank, Palestine: Nablus, Jenin, Tubas, Tulkarm, Salfeit, Qalqilya, Ramallah, Jericho, Jerusalem, Bethlehem and Hebron (Fig. 1).

The Institutional Review Board (IRB) at An-Najah National University approved the aims of this study, its protocols, and the informed consent forms; IRB archived number 9/April/2016. This study was carried out according to the requirements of the declarations of Helsinki.

The study was carried out using the method of interviews with herbalists, traditional healers, hairdressers and rural dwellers that used herbal remedies in the treatment of different types of hair and scalp disorders and for the alteration of their aesthetic appearance. These informants represented most of the practitioners in this field in the West Bank (Socio-demographic characteristics are presented in Table 1).

**Table 1:** Socio-demographic characteristics of the study population (*N* = 267)

Variable	n (%)^a^
Age (year)	
16–29	129 (48.3)
30–39	67 (25.1)
40–49	50 (18.7)
50–59	17 (6.4)
≥ 60	4 (1.5)
Education	
Illiterate level	6 (2.2)
Elementary or preparatory level	23 (8.6)
Secondary level	70 (26.2)
University level	168 (62.9)
Income	
Low	14 (5.2)
Medium	229 (85.8)
High	24 (9.0)
Material status	
Single	115 (43.1)
Married	144 (53.9)
Others (divorced or widow)	8 (3.0)
Place of residence	
City	116 (43.4)
Village	145 (54.3)
Refugee camp	6 (2.2)
Knowledge of natural products	
Media (TV, Radio, Journals...etc)	46 (17.2)
Relatives and friends	108 (40.4)
Attarine (herbalists)	11 (4.1)
Physicians	2 (0.7)
Pharmacists	11 (4.1)
Internet	88 (33.0)
Other sources	1 (0.4)
Obtainment of the natural products	
Attarine (herbalists)	194 (72.7)
Pharmacies	32 (12.0)
Friends	3 (1.1)
Wild and nature	38 (14.2)

^a^Data are presented as frequency (percent) from the total population studies (*N* = 267)

A sufficient sample of herbalists and cosmetic practitioners, from various regions, were met by researchers and asked to answer a face-to-face questionnaire. They were interviewed in Arabic after getting their verbal consent only once.

Questionnaires were administered through personal contact discussions. This method is an effective and easy option for data collection. The objective of this survey was to obtain information on several issues including: (i) the names of plant, mineral and animal products commonly used as cosmeceuticals for the hair and scalp, (ii) the type of aesthetic purpose or disorder treated by these natural products, (iii) the methods of preparing and (iv) parts used for cosmeceutical treatment. For the aim of obtaining clear information, names of plants or other natural products were translated later into English and Latin. In most cases, the interviews often started in the form of informal discussions to obtain the confidence of the interviewees.

All of the 41 plants and 10 other natural products, minerals and animals products, were collected from the interviewees (herbalists, traditional healers, hairdressers and rural dwellers) and kept in special glass frames and later identified by the pharmacognosist Dr. Nidal Jaradat. The identity of each plant species mentioned by the interviewees was confirmed and verified by using photographs and live specimens. A medicinal use was accepted as valid only if it was mentioned by at least three independent herbal practitioners. Samples of these collected herbs were given a herbarium specimen number as shown in Table 2 and voucher samples were kept at the Pharmacognosy Laboratory of the Department of Pharmacy, Faculty of Medicine and Health Sciences at An-Najah National University.

**Table 2:** Herbals used in the treatment of hair and scalp in the West Bank/Palestine

Plant names (Latin, English and Arabic names) with their voucher specimen codes	Family	Citations	Use value
*Allium cepa* L./ Onion/بصل/ IPharm-PCT-2703	Amaryllidaceae	34	0.13
*Allium sativum* L./ Garlic/ثوم/Pharm-PCT-2704	Amaryllidaceae	156	0.58
*Nigella arvensis* L./ Nigella/ قزحه/ Pharm-PCT-1640	Ranunculaceae	28	0.10
*Petroselinum crispum* (Mill.) Fuss/Parsley/ بقدونس/ Pharm-PCT-2739	Apiaceae	8	0.03
*Pimpinella anisum* L./Anise/اليانسون /Pharm-PCT-2768	Apiaceae	38	0.14
*Cocos nucifera* L./ Coconut/جوز الهند/Pharm-PCT-2764	Arecaceae	84	0.31
*Barbarea vulgaris* R.Br./ Rocket cress/ جرجير/Pharm-PCT-2757	Brassicaceae	15	0.06
*Raphanus raphanistrum subsp. sativus* (L.) Domin / Fodder Radish/فجل/Pharm-PCT-2007	Brassicaceae	4	0.01
*Anthemis cotula* L./ Chamomile/البابونج/ Pharm-PCT-178	Compositae	46	0.17
*Cucumis sativus* L./ Cucumber/خيار/ Pharm-PCT-2737	Cucurbitaceae	18	0.07
*Citrullus colocynthis* (L.) Schrad. / Bitter Apple/ حنظل/ Pharm-PCT-628	Cucurbitaceae	8	0.03
*Ricinus communis* L./ Castor/خروع/Pharm-PCT-2742	Euphorbiaceae	173	0.65
*Salvia fruticosa* Mill./Sage/مريمية/Pharm-PCT-2117	Lamiaceae	16	0.06
*Origanum syriacum* L./Syrian oregano(Thyme)/زعتر/Pharm-PCT-1496	Lamiaceae	24	0.09
*Rosmarinus officinalis* L./Rosemary/اكليل الجبل/Pharm-PCT-2732	Lamiaceae	9	0.03
*Mentha longifolia* (L.) L./Mint/نعنع/Pharm-PCT-1566	Lamiaceae	4	0.01
*Lavandula coronopifolia* Poir./Lavender/خزامي/ Pharm-PCT-1367	Lamiaceae	4	0.01
*Persea americana* Mill./ Avocado/ الافوكادو/Pharm-PCT-2740	Lauraceae	23	0.09
*Trigonella arabica* Delile/ Fenugreek/حلبه/Pharm-PCT-2511	Leguminosae	64	0.24
*Lawsonia inermis* L./ Henna/حناء/ Pharm-PCT-2736	Lythraceae	223	0.84
*Punica granatum* L./ Pomegranate/رمان/ Pharm-PCT-2721	Lythraceae	28	0.10
*Hibiscus sabdariffa* L./ Roselle/كركديه/ Pharm-PCT-2752	Malvaceae	9	0.03
*Abelmoschus esculentus* (L.) Moench/ Okra/باميه/ Pharm-PCT-2772	Malvaceae	10	0.04
*Azadirachta indica* A.Juss./ Neem/نيم/Pharm-PCT-2769	Meliaceae	6	0.02
*Musa paradisiaca* L./ Banana/موز/ Pharm-PCT-2715	Musaceae	26	0.10
*Myristica fragrans* Houtt./ Nutmeg/جوزة الطيب/Pharm-PCT-2716	Myristicaceae	7	0.03
*Melaleuca alternifolia* (Maiden & Betche) Cheel / Tea tree oil/شجرة الشاي/Pharm-PCT-2765	Myrtaceae	6	0.02
*Syzygium aromaticum* (L.) Merr. & L.M.Perry/ Clove/كبش قرنفل/ Pharm-PCT-2767	Myrtaceae	22	0.08
*Olea europaea* L. / Olive/الزيتون/ Pharm-PCT-1664	Oleaceae	369	1.38
*Sesamum indicum* L./ Sesame/سمسم/Pharm-PCT-2722	Pedaliaceae	35	0.13
*Prunus dulcis* (Mill.) D.A.Webb/ Almond/ لوز/Pharm-PCT-143	Rosaceae	76	0.28
*Malus domestica* Borkh./Apple vinegar/خل التفاح/Pharm-PCT-2766	Rosaceae	118	0.44
*Rosa canina* L./Rose/الورد الجوري/ Pharm-PCT-2052	Rosaceae	4	0.01
*Citrus limon* (L.) Osbeck /Lemon/ لمون/ Pharm-PCT-2741	Rutaceae	82	0.31
*Aegle marmelos* (L.) Corrêa /Quince/سفرجل/Pharm-PCT-2702	Rutaceae	11	0.04
*Simmondsia chinensis* (Link) C.K. Schneid. /Jojoba/جوجوبا/Pharm-PCT-2771	Simmondsiaceae	5	0.02
*Capsicum frutescens* L./ Chili pepper/ الفليفلة الشجيرية/ Pharm-PCT-2760	Solanaceae	6	0.02
*Camellia sinensis* (L.) Kuntze/ Green tea/شاي اخضر/Pharm-PCT-2706	Theaceae	32	0.12
*Urtica pilulifera* L./ Nettle/قريص/Pharm-PCT-2561	Urticaceae	11	0.04
*Aloe vera (L.) Burm.f. /Aloe/ صبار/Pharm-PCT-115*	Xanthorrhoeaceae	85	0.32
*Zingiber officinale Roscoe/Ginger/زنجبيل/Pharm-PCT-2724*	Zingiberaceae	14	0.05

### Data analysis

Statistical analyses were performed by using the Statistical Package for Social Sciences (SPSSversion17.0).

All citations were placed into ailment categories for each type of cancer. The factor of informant’s consensus (F_ic_) was employed to indicate how homogenous the information was. In fact, its main use is to select the disease categories where there is consensus on the use of plants among the informants. The F_ic_ value is close to 0 if plants are chosen randomly or if informants do not exchange information about their use. High values of F_ic_ (close to 1) occur when there is a well-defined selection criterion in the community and/or if information is frequently exchanged between informants [19].

The F_ic_ is calculated as in the following equation:

$$ {F}_{ic}=\frac{Nur- Nt}{Nur-1} $$

Where Nur is the number of use citations in each category and Nt is the number of taxa used.

Fidelity level (Fl) was defined as the ratio between the number of informants who independently suggested the use of a species for the same major purpose and the total number of informants who mentioned the plant for any use. Fl is of equal importance to F_ic_ and it can be calculated according to the following equation*:*

$$ Fl={\frac{ N p}{N}}^{\ast }100 $$

Where Np is the number of informants that reported a use of a plant species to treat a particular disease and N is the number of informants that used the plants as a medicine to treat any given disease.

The use-value (UV) is a quantitative method that can be used in order to prove the relative importance of species known locally. It can be calculated according to the following equation:

$$ \mathrm{UV}=\frac{\sum U}{n} $$

Where UV is the use value of a species; *U* is the number of citations per species; *n* is the number of informants.

Results of calculated F_IC_, Fl, and UV are shown in Tables 2, 3, 4 and 5.

## Results

Table 1 summarizes the Socio-demographic characteristics of our sample of 267 women included in the study. Respondents belong to all age groups but mostly (48.3%) were 16–29 years of age. They are from various educational backgrounds with the majority of them (62.9%) being from university-educated backgrounds, whilst the minority (10.8%), were from an elementary level of education or illiterate. This ratio of illiterate versus university graduated women, who participated in this study, sounds consistent with the most recent status of education in Palestine. We also sampled across income (85.8% were of medium income) and marital status (53.9% married women, 43.1% single and 3% were divorced or widowed). Knowledge of natural products was also investigated; most of them obtained their information from relatives and friends (40.4%), the internet (33.0%). Most of them (72.7%) obtained the natural products they used from “Attarine;” merchants who sell herbs and natural products.

### Diversity of plants traditionally used in local cosmetics

**Fig. 2:**
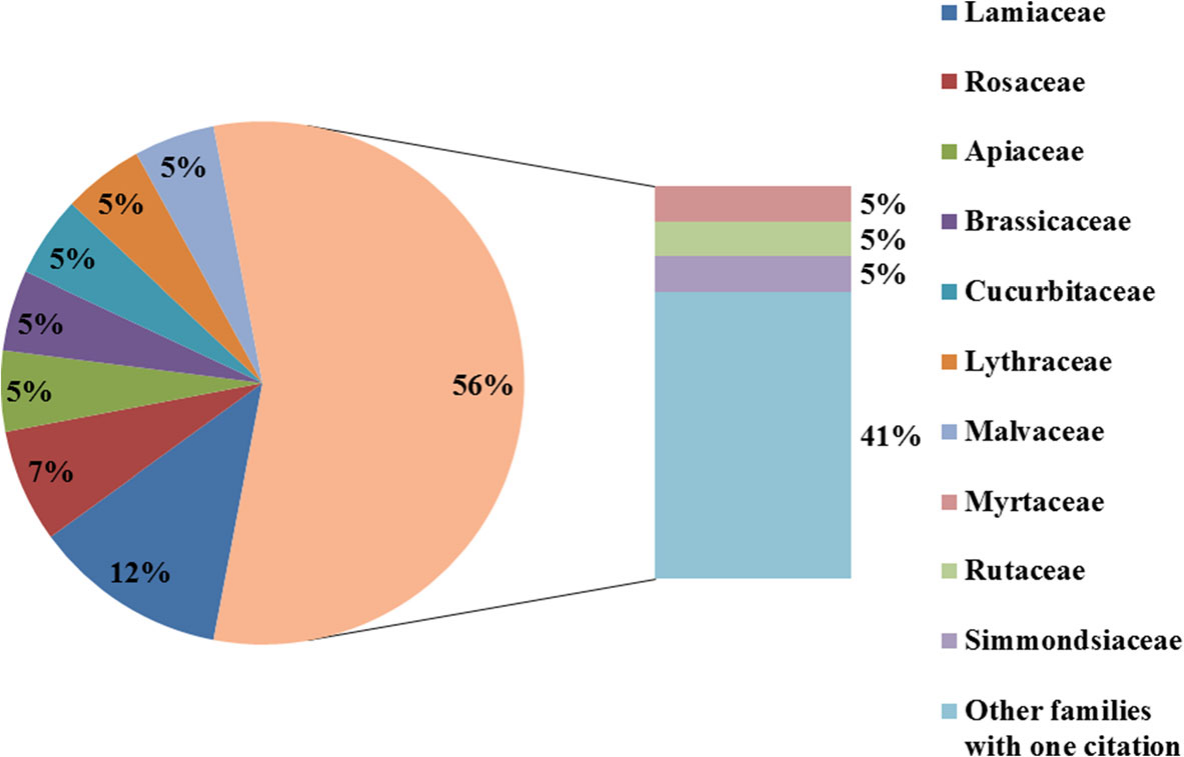
Family use value for the used plants

In total, 41 plant species distributed across 40 genera and 27 families were reported as locally and traditionally used cosmetics and cosmeceuticals for the hair and scalp in Palestine, which were presented in Table 2 and the family use value is explained in Fig. 2. The largest families of cosmetic plants were Lamiaceae and Rosaceae (5 species and 3 species respectively), with only 1–2 species reported for the 19 remaining families. However, the calculated UV values for plants that belong to Lamiaceae family are close to 0 whilst only *Rosa canina,* which belongs to Rosaceae, showed similar UV value. In contrast, the highest UV value (0.84) was registered for *Lawsonia inermis,* which belongs to the Lythraceae family.

### Traditional treatment of hair and scalp disorders using animal and/or mineral extracts

Similarly to the above-mentioned disorders, hair loss, split hair endings and hair damage were widely mentioned with a Fic equal to 0.94. However, the highest Fic (1) was recorded for the enhancing of hair color.

### Traditional preparation method of the natural Cosmeceuticals for hair and scalp disorders

**Fig. 3:**
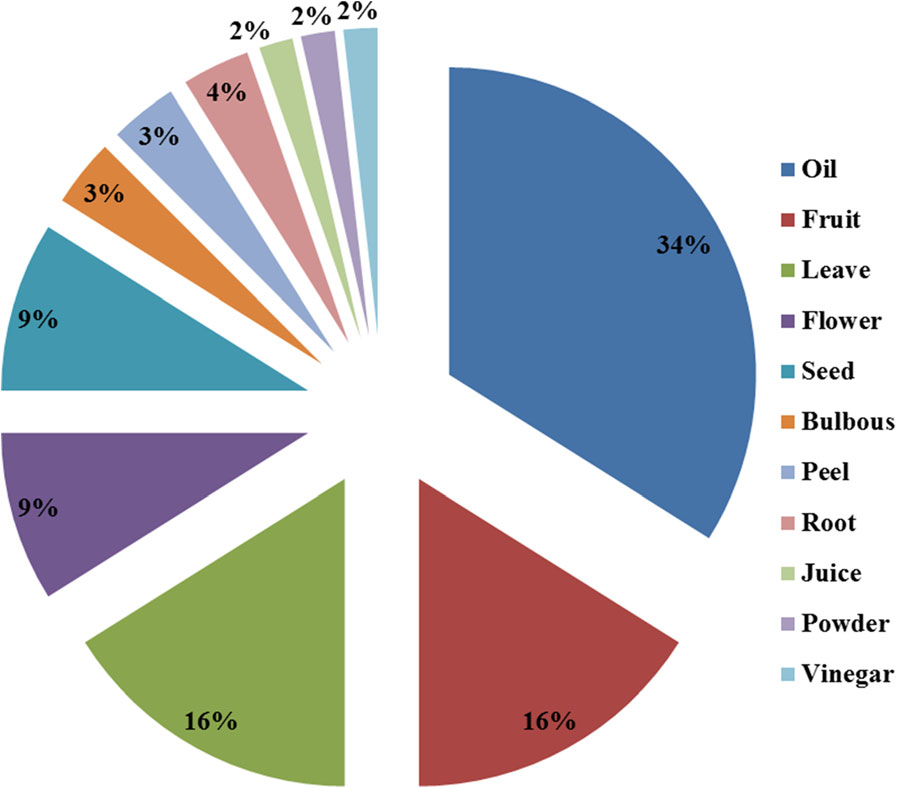
The parts used of herbals in cosmetics

Table 6 and Fig. 3 summarized the parts used as well as the method of preparation and application of herbal products to the hair and scalp. As can be seen, oils alone or oil mixtures are the most used products and they are applied or rubbed directly onto the scalp. In addition, olive oil was used in most herbal products, not only as a vehicle but also because of its emollient effect and high nutritional value. This showed synergistic effects in most of the listed treatments such as: hair endings, damaged hair and hair loss. At a lower degree, we found that *Ricinus communis* oil showed synergistic effects with olive oil in most of the reported treatments, especially in the treatment of split hair endings, hair loss and the use of hair conditioners (Table 3). Regarding products obtained from mineral or animal sources, Table 7 showed that the most frequently used methods in the use of these products were by mixing them with other natural products such as: eggs, honey or olive oil. Olive oil was again used as a vehicle but to a lesser extent than in herbal products. However, the egg was used in about 50% of the animal and mineral products. This may be explained due to its high lecithin content and nutritional value (Table 4). In addition, it was used with honey in the treatment of 9 out of the 13 reported hair and scalp disorders. They showed synergistic effects in 6 out of these 9 treatments, especially for the treatment of split hair endings and hair loss (Table 5). Meanwhile, Table 8 showed the Factor of Informant’s Consensus (Fic) values for minerals and animals products, categorized by the types of cosmeceutical treatment.

**Table 3:** Plants used as home remedies for treatment of hair and hair scalp in the West Bank/Palestine

Plant name	Hair endings	Hair damage	Hair loss	Baldness	Dandruff	Lice	Scalp acne	Alopecia areata	Scabies	Enhance the color of the hair	Hair dyes	Hair cleaners	Hair conditioners
*Aloe vera*	20.0	13.0	13.0	6.0	14.0	-	-	6.0	-	6.0	-	-	7.0
FL	23.5	15.3	15.3	7.1	16.5			7.1		7.1			8.2
*Prunus dulcis*	24.0	8.0	25.0	-	7.0	-	8.0	-	-	-	-	4.0	-
FL	31.6	10.5	32.9		9.2		10.5					5.3	
*Cocos nucifera*	16.0	14.0	10.0	4.0	-	-	-	-	-	9.0	-	-	31.0
FL	19.0	16.7	11.9	4.8						10.7			36.9
*Camellia sinensis*	-	-	5.0	11.0	-	5.0	-	-	-	11.0	-	-	-
FL			15.6	34.4		15.6				34.4			
*Melaleuca alternifolia*	-	-	-	-	-	6.0							
FL						100.0							
*Capsicum frutescens* L.	-	-	-	-	-	-	-	6.0	-	-	-	-	-
FL								100.0					
*Urtica pilulifera*	6.0	-	5.0	-	-	-	-	-	-	-	-	-	-
FL	54.5		45.5										
*Malus domestica*	11.0	-	6.0	5.0	30.0	31.0	8.0	9.0	4.0	-	6.0	8.0	-
FL	9.3		5.1	4.2	25.4	26.3	6.8	7.6	3.4		5.1	6.8	
*Persea americana*	10.0	6.0	-	-	-	-	-	-	-	-	-	-	7.0
FL	43.5	26.1											30.4
*Olea europaea* L.	60.0	48.0	55.0	20.0	53.0	5.0	7.0	9.0	-	13.0	-	30.0	69.0
FL	16.3	13.0	14.9	5.4	14.4	1.4	1.9	2.4		3.5		8.1	18.7
*Ricinus communis*	43.0	22.0	40.0	22.0	15.0	-	-	6.0	-	-	-	-	25.0
FL	24.9	12.7	23.1	12.7	8.7			3.5					14.5
*Barbarea vulgaris*	4.0	-	11.0	-	-	-	-	-	-	-	-	-	-
FL	26.7		73.3										
*Trigonella arabica*	7.0	-	11.0	12.0	7.0	-	23.0	-	-	-	-	-	4.0
FL	10.9		17.2	18.8	10.9		35.9						6.3
*Zingiber officinale*	-	-	5.0	4.0	5.0	-	-	-	-	-	-	-	-
FL			35.7	28.6	35.7								
*Sesamum indicum*	-	11.0	8.0	-	4.0	-	-	-	-	4.0	-	-	8.0
FL		31.4	22.9		11.4					11.4			22.9
*Lawsonia inermis*	10.0	5.0	5.0	-	9.0	-	9.0	-	10.0	40.0	129.0	-	6.0
FL	4.5	2.2	2.2		4.0		4.0		4.5	17.9	57.8		2.7
*Nigella arvensis*	-	-	12.0	-	5.0	4.0	-	7.0	-	-	-	-	-
FL			42.9		17.9	14.3		25.0					
*Musa paradisiaca*	6.0	-	-	-	-	-	-	-	-	-	-	-	20.0
FL	23.1												76.9
*Petroselinum crispum*	-	-	-	-	-	8.0	-	-	-	-	-	-	-
FL						100.0							
*Punica granatum* L.	-	4.0	12.0	-	-	-	-	-	5.0	7.0	-	-	-
FL		14.3	42.9						17.9	25.0			
*Salvia fruticosa*	6.0	-	-	-	-	-	-	-	-	6.0	-	4.0	-
FL	37.5									37.5		25.0	
*Origanum syriacum*	-	-	15.0	-	9.0	-	-	-	-	-	-	-	-
FL			62.5		37.5								
*Pimpinella anisum*	-	-	-	-	-	38.0	-	-	-	-	-	-	-
FL						100.0							
*Citrus limon*	-	-	7.0	4.0	43.0	10.0	14.0	-	-	-	4.0	-	-
FL			8.5	4.9	52.4	12.2	17.1				4.9		
*Syzygium aromaticum*	-	8.0	-	-	-	-	14.0	-	-	-	-	-	-
FL		36.4					63.6						
*Allium cepa*	-	-	4.0	9.0	-	6.0	-	15.0	-	-	-	-	-
FL			11.8	26.5		17.6		44.1					
*Anthemis cotula*	-	-	-	-	10.0	4.0	-	-	4.0	17.0	5.0	6.0	-
FL					21.7	8.7			8.7	37.0	10.9	13.0	
*Allium sativum*	-	-	16.0	38.0	-	9.0	12.0	62.0	14.0	-	-	-	5.0
FL			10.3	24.4		5.8	7.7	39.7	9.0				3.2
*Hibiscus sabdariffa*.	-	-	-	-	-	-	-	-	-	9.0	-	-	-
FL										100.0			
*Rosmarinus officinalis*	-	-	-	-	-	-	-	9.0	-	-	-	-	-
FL								100.0					
*Cucumis sativus*	-	-	-	-	-	18.0	-	-	-	-	-	-	-
FL						100.0							
*Aegle marmelos*	-	-	-	-	7.0	-	-	-	-	-	-	4.0	-
FL					63.6							36.4	
*Raphanus raphanistrum*	-	-	-	-	-	-	-	4.0	-	-	-	-	-
FL								100.0					
*Mentha longifolia*	-	-	-	-	-	-	-	-	-	-	-	-	4.0
FL													100.0
*Azadirachta indica*	-	-	-	-	-	6.0	-	-	-	-	-	-	-
FL						100.0							
*Myristica fragrans*	-	-	-	-	-	-	7.0	-	-	-	-	-	-
FL							100.0						
*Rosa canina*	-	-	-	-	-	-	4.0	-	-	-	-	-	-
FL							100.0						
*Citrullus colocynthis*	-	-	-	-	-	-	-	8.0	-	-	-	-	-
FL								100.0					
*Simmondsia chinensis*	-	-	-	-	-	-	-	-	-	-	-	5.0	-
FL												100.0	
*Lavandula coronopifolia*	-	-	-	-	-	-	-	-	-	-	-	4.0	-
FL												100.0	
*Abelmoschus esculentus*	-	-	-	-	-	-	-	-	-	-	-	-	10.0
FL													100.0

**Table 4:** Factor of informant’s consensus (F_ic_) for herbals, categorized by the types of cosmeceutical treatment

Problem	Total uses	No. of plant	Fic
Hair endings	223	13	0.95
Hair damage	139	10	0.93
Hair loss	265	19	0.93
Bladness	135	11	0.93
Dandruff	218	14	0.94
Lice	150	13	0.92
Scalp acne	106	10	0.91
Alopecia areata	141	11	0.93
Scabies	37	5	0.89
Enhance the color of the hair	122	10	0.93
Hair dyes	144	4	0.98
Hair cleaners	65	8	0.89
Hair conditioners	196	12	0.94

**Table 5:** Fidelity level of other natural products used in the treatment of hair and scalp in the West Bank/Palestine

Animal Product	Hair ending	Hair damage	Hair loss	Baldness	Dandruff	Lice	Scalp acne	Alopecia areata	Scabies	Enhance hair color	Hair dyes	Hair cleaners	Hair conditioners
Egg	28.0	24.0	30.0	10.0	10.0	-	-	7.0	-	-	-	-	21.0
FL	21.5	18.5	23.1	7.7	7.7			5.4					16.2
Honey	15.0	6.0	17.0	9.0	5.0	5.0	9.0	26.0	-	-	-	-	5.0
FL	15.5	6.2	17.5	9.3	5.2	5.2	9.3	26.8					5.2
Yogurt	7.0	12.0	-	4.0	-	-	-	-	-	3.0	-	-	6.0
FL	21.9	37.5		12.5						9.4			18.8
Fish oil	4.0	-	-	-	-	-	-	-	-	-	-	-	-
FL	100.0												
Milk	-	7.0	4.0	4.0	-	-	-	-	-	-	-	-	-
FL		46.7	26.7	26.7									
Salt	-	-	-	-	4.0	-	-	-	-	-	-	-	5.0
FL					44.4								55.6
Butter	-	-	4.0	-	-	-	-	-	-	-	-	-	5.0
FL			44.4										55.6
Kerosene	-	-	-	-	-	17.0	-	-	-	-	-	-	-
FL						100.0							
Mustard	-	-	-	-	-	-	-	5.0	-	-	-	-	-
FL								100.0					
Mineral oil	-	-	-	-	-	7.0	-	-	-	-	-	-	-
FL						100.0

**Table 6:** Plant products, parts used and preparation methods

Plant name	Parts used	Preparation methods
*Aloe vera*	Oil	Apply to the hair alone or with olive and other oils (like: sesame, avocado, almond and castor oil).
	Leave	Boil the leaves and apply to hair
*Prunus dulcis*	Oil	Apply to hair by rubbing
*Cocos nucifera*	Oil	Apply to hair by rubbing.
		Can be diluted with other oils (olive, sesame, castor) and applied to hair.
*Camellia sinensis*	Leaves	Soak in warm water and apply.
*Melaleuca alternifolia*	Oil	Apply to hair by rubbing.
*Capsicum frutescens*	Fruit	Smashed and mixed with olive oil and vinegar and applied topically.
*Urtica pilulifera*	Leaves	Smashed and mixed with olive oil and applied topically.
*Malus domestica*	Vinegar	Dilute with water and apply, can mix with olive oil before its application.
*Persea americana*	Oil	Apply to hair by rubbing (can mix with other oils like, olive, coconut, aloe).
	Fruit	Smash the fruit and mix with olive oil and apply topically.
*Olea europaea*	Oil	Apply by rubbing.
		Prepare in soap and use for cleansing.
*Ricinus communis*	Oil	Apply by rubbing.
		Dilute with olive oil and rube.
*Barbarea vulgaris*	Oil	Soak in water and apply to the hair
	Leave	
*Trigonella arabica*	Oil	Apply the oil by rubbing after mixing with mustard and olive oil.
	Seed	Soak the seed in warm water blend and apply
*Zingiber officinale*	Root	Smash the roots boil in water and apply
	Oil	
*Sesamum indicum*	Oil	Apply by rubbing, can mix with other oils (Castor, olive and coconut)
*Lawsonia inermis*	Leave	Mill the leaves, then knead the powder with warm water and apply for 30 min, then wash
	Powder	Apply after kneading with warm water and green tea
		Apply after kneading with warm water
*Nigella arvensis*	Oil	Apply the oil by rubbing after mixing with honey and watercress and olive oils
	Seed	Mill the seed, soak in warm water and mix with watercress then apply
*Musa paradisiaca*	Fruit	Smashed, boiled for 10 min and mixed with olive oil then applied to the hair
	Peel	
*Petroselinum crispum*	Leave	Boil in water and apply
	Seed	
*Punica granatum*	Fruit	Squeeze and apply
	Peel	Mill the peel, soak with warm water and apply
*Salvia fruticosa*	Leave	Boil in water and apply
	Flower	
*Origanum syriacum*	Leave	Boil in water and apply
	Oil	Apply the oil by rubbing, also can be mixed with other oils (olive, castor and avocado)
*Pimpinella anisum*	Oil	Apply by rubbing, can mix with olive and castor oil
*Citrus limon*	Fruit	Squeeze the fruit then mix the juice with olive oil and apply
	Juice	
*Syzygium aromaticum*	Oil	Apply by rubbing, can mix with olive oil.
	Flower	Mill and soak with water and mix with garlic then apply.
*Allium cepa*	Bulbous	Squeeze mix with ginger and oils (olive, caster and aloe) then apply
*Anthemis cotula*	Flower	Boil in water and apply
*Allium sativum*	Bulbous	Smash the garlic mix with yogurt and apply
	Oil	Apply the oil by rubbing
*Hibiscus sabdariffa*	Flower	Soak in warm water and apply to hair
*Rosmarinus*	Leave	Boil in water and apply
*Cucumis sativus*	Fruit	Squeeze and mix with oil and apply
*Aegle marmelos*	Fruit	Boil in water blend and apply
	Seed	Mill the seed and soak in warm water then apply
*Raphanus raphanistrum*	Root	Squeeze and blend with other oils (castor, olive)
*Mentha longifolia*	Oil	Apply the oil by rubbing, can be diluted with olive oil
	Leave	Boil the leaves and wash the hair 1 h before bath
*Azadirachta indica*	Oil	Apply to hair by rubbing
*Myristica fragrans*	Seed	Soak in water blend and apply, can mix with oils
*Rosa canina*	Flower	Apply by rubbing
*Citrullus colocynthis*	Fruit	Blend and apply
*Simmondsia chinensis*	Oil	Mix with olive, watercress and castor oil then apply by rubbing
*Lavandula coronopifolia*	Oil	Mix with olive, watercress and castor oil then apply by rubbing
*Abelmoschus esculentus*	Fruit	Boil in water and apply to the hair

**Table 7:** Minerals and animal products and their preparation methods

Name	Preparation methods
Egg	Mix with olive oil and garlic then apply.
	Mix egg yolk with almond oil and apply by rubbing.
	Mix with honey and Turmeric and apply.
Honey	Dilute with water and apply by rubbing.
	Mix with egg and Turmeric and apply by rubbing.
Yogurt	Apply by rubbing.
	Mix with egg and apply by rubbing.
Fish oil	Dilute with olive oil and apply by rubbing.
	Mix with egg and apply
Milk	Mix with Turmeric and honey then apply by rubbing.
Salt	Mix with water and apply.
Butter	Mix with egg and apply
Kerosene	Apply by rubbing.
Mustard	Mix with honey and apply by rubbing.
Mineral oil	Mix with olive oil and apply by rubbing

**Table 8:** Factor of informant’s consensus (F_ic_) for minerals and animals products, categorized by the types of cosmeceutical treatment

Problem	Nur	Nt	Fic
Hair endings	54	4	0.94
Hair damage	49	4	0.94
Hair loss	55	4	0.94
Bladness	27	4	0.88
Dandruff	19	3	0.89
Lice	29	2	0.96
Scalp acne	9	1	1.00
Alopecia areata	38	3	0.95
Scabies	0	0	0.00
Enhance the color of the hair	3	1	1.00
Hair dyes	0	0	0.00
Hair cleaners	0	0	0.00
Hair conditioners	42	5	0.90

**Table 9:** Summarizes published cosmeceutical and skin uses of these frequently used plants

Plant species	Reported ethnopharmacological use with reference source	Cosmeceutical and skin uses with reference source	Side effects and toxicity with reference source
*Olea europaea*	Reported usage in ethnomedicine in Palestine [23], Italy [24], Spain, and other Mediterranean areas [25].	To prevent hair loss and skin cleanser [26].	Leaves cause hepato-cellular and renal abnormality [27].
*Lawsonia inermis*	Reported in Africa [28], Southern Asia specially India [29], Palestine [30], and worldwide [31].	Coloring material, fungicidal and anti-inflammatory [32].	It may cause loss of body balance, and paralysis [33].
*Ricinus communis*	Reported in India [23]	Reported its anti-inflammatory [34], antimicrobial and antifungal activities [35].	No significant toxic effects [36].
*Allium sativum*	Reported in developing countries [37]	Reported as antiseptic and expectorant [38]	Reported to have toxic potential, with a demonstrated capability to alter biochemical indices in vital tissues [39]
*Aloe vera*	Reported in southern Africa [40], Nigeria [41], Mediterranean countries [42], Asia [43] and India [44]	Reported in treatment of dry skin, improve the skin integrity, decrease appearance of acne wrinkle and decrease erythema [45]	Overdose reported to lead to colicky abdominal spasms and pain, as well as the formation of thin, watery stools [46]
*Cocos nucifera*	Reported in Southeast Asia [47], India [48], Africa [49], and American continent [50].	Reported as Anti-bacterial, antifungal [51], preventing hair loss, wound healing, and dermatitis [52], .	Low toxicity effect reported [53].
*Trigonella arabica*	Reported in India, Africa [54], and Egypt [55].	Treatment of inflammation [56]	Administration at higher dose induced toxicity including teratogenic, foetotoxic, reproductive changes and the abnormal shapes of the sperms [57]
*Prunus dulcis*	Reported in Iraq [58] and Lebanon [59] and well known Worldwide	High antioxidant activity [60] and used for premature hair fall [61]	No reference
*Citrus limon*	Reported throughout the world [62]	Skin care and anti-oxidants [62].	No reference
*Pimpinella anisum*	Reported in Mediterranean Region [63], Palestine [64], the Middle East [65] and Spain [63]	Reported to have insecticidal effect [66], antioxidant and anti-inflammatory effect [67],	No reference
*Punica granatum*	Reported in India [68], Algeria [69], Africa [70], America [71], Spain [72].	Evidence for in vitro assay activity as antioxidant [73] and protects against the adverse effects ultraviolet radiation [74, 75].	Considered as non-toxic [76, 77]
*Sesamum indicum*	Reported in India [78], USA [79], Pakistan [80], Africa [81], China [82] and Sudan [83].	Reported to have antioxidant activity [84] and wound healing [85].	Practically non-toxic [86].

### Traditional treatment of hair and scalp disorders using plants extracts

In Table 4, reported hair loss, dandruff, split hair endings and lice treatment were reported as the most treated disorders. In fact, the total number of plant species used was 19, 14, 13 and again 13 with a factor of informant’s consensus (Fic) for these disorders corresponding to 0.93, 0.94, 0.95, and 0.92 respectively.

## Discussion

Several studies have shown that around 80% of rural populations, in developing countries, consider herbal remedies as significant and important. It has been shown, in recent years, that the use of natural herbal products, has increased in both developed and developing countries, which is due to many varying, reasons [20, 21].

As shown in Table 1, most of the respondents have a high level of education, with the majority of them (62.9%) graduating from University. The same table also showed that the majority of respondents (72.7%) usually obtain their natural products from “Attarines”, which indicates a high level of trust in these folkloric venders. It also showed that Pharmacists, who are believed to be the group of professional people with the appropriate knowledge and educational background to answer correctly about the safety and efficacy of these products, were rarely contacted regarding the use of natural products. This may imply that there is a lack of trust in these healthcare professionals or that a belief system exists that suggests that these products can be dealt with traditionally; the use of “Attarines,” and that, due to their long- standing use, these natural products are considered to be safe.

In Palestine, natural cosmeceutical and nutraceutical products are frequently commercialized in herbal shops and, at a lower percentage, in Community Pharmacies and are also, often, prepared domestically. The knowledge and the attitude that people have towards domestic home cosmeceutical and nutraceutical remedies are mainly based on their traditional, folkloric uses as well as updated information. This information is generally obtained from friends’ and/ or relatives as well as from the Internet. Tables 2 and 6 show the natural cosmeceutical remedies, conventionally used for decorating hair and/ or the healing of hair and scalp disorders in the studied area. Plant-derived home-made cosmetics, cosmeceuticals and remedies for hair and scalp disorders include, approximately, 56 preparation methods coming from an average of 41 botanical species, whilst remedies derived from minerals or animals are listed in Tables 2 and 6.

### Folk cosmetics and aesthetic values amongst Palestinian population

Cosmetic preparations used for the: coloring or lightening of hair, prevention of hair loss or the treatment of scabies, have been included in our study. Most of the plant ingredients (*Anthemis cotula, Rosa canina*, *Simmondsia chinensis*, *Lavandula coronopifolia*, and *Prunus dulcis*) are also used today in modern phyto-cosmetics, whilst a few ingredients are less known, nowadays, for cosmetic purposes (*Citrullus colocynthis*, *Abelmoschus esculentus* and *Aegle marmelos*). From a cultural point of view, it is interesting to note how Palestinian women are still trying to find a diverse and wide range of ingredients in order to enhance their hair appearance; this usually involves the lightening or coloring of their hair to a shade of blond, which is considered a synonym of beauty for most of them. The use of chamomile paste, apple vinegar, and lemon juice indicates a new trend in the cultural concepts of aesthetics, which has been emphasized in recent years. Nowadays, in fact, blond hair, altered by cosmetic products, is normally considered a sign of beauty in Arab countries. Therefore, many women are willing to add and use any natural additives to enhance the performance of certain hair dyes such as Henna and Chamomile.

### Ethnocosmeceuticals

Many reported formulations have been or are still being used to enhance the appearance of hair: lightening and coloring, prevention of hair loss and to combat scabies. Less is known about the phyto-pharmacology of the ingredients used in these preparations, they often had an emollient rule and were thought to optimize and restore the functions of the scalp and appendices, which are highly affected by a lifestyle characterized by hard daily activities. This group of remedies comprises onions, honey, eggs, aloe, fish oil, mustard, rosemary, pomegranate, and even milk. A few of these species are in fact medicinal plants, well-known in the modern, evidence-based European and also Mediterranean and Arab phytotherapy herbal treatise of the past five centuries [22].

### Ingredients of animal and mineral origin

Bee products (wax and honey), milk products (yogurt, fresh milk, and butter), eggs and even kerosene represent the most commonly reported ingredients of non herbal origin used cosmetically by rural women (Table 2). A few of these ingredients were used as excipients and active ingredients at the same time, mainly as emollients, whilst others, such as kerosene, were used as pesticides.

### Historical considerations

Similar to other studies conducted on home cosmeceutical remedies, we can deduct that traditional Palestinian knowledge, in the preparation of domestic home remedies and cosmeceutical products for the healing of hair and scalp disorders, rarely include exotic or expensive ingredients from the Mediterranean market. The only exception to this is represented by the use of oils extracted from *Simmondsia chinensis*, *Lavandula coronopifolia*, *Pimpinella anisum*, *Syzygium aromaticum*, and *Azadirachta indica* oils. Thus the Palestinian cosmetic and cosmeceutical practices have taken a different direction from the historical “schools” of cosmetics.

According to our findings, members of the Lamiaceae and Rosaceae families were the most commonly used cosmeceuticals as reported in Table 2. The methods used in the preparation of these herbal products consisted of the mixing of more than one natural product together followed by the direct application of this mixed product to the hair or scalp. This method, however, may result in low compliance as these products are often oily or have bad organoleptic properties, making them difficult to be clean without the use of strong detergents, which may, possible, have a detrimental effect on the hair and scalp. Asking for the appropriate advice and counseling from a pharmacist may resolve this issue. Pharmacists are capable of preparing these products in a more suitable form such as creams or lotions, which are easily rinsed from the hair whilst leaving a suitable odor.

**Fig. 4:**
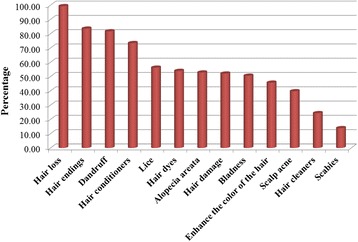
Hair and scalp disorders treated by herbals

**Fig. 5:**
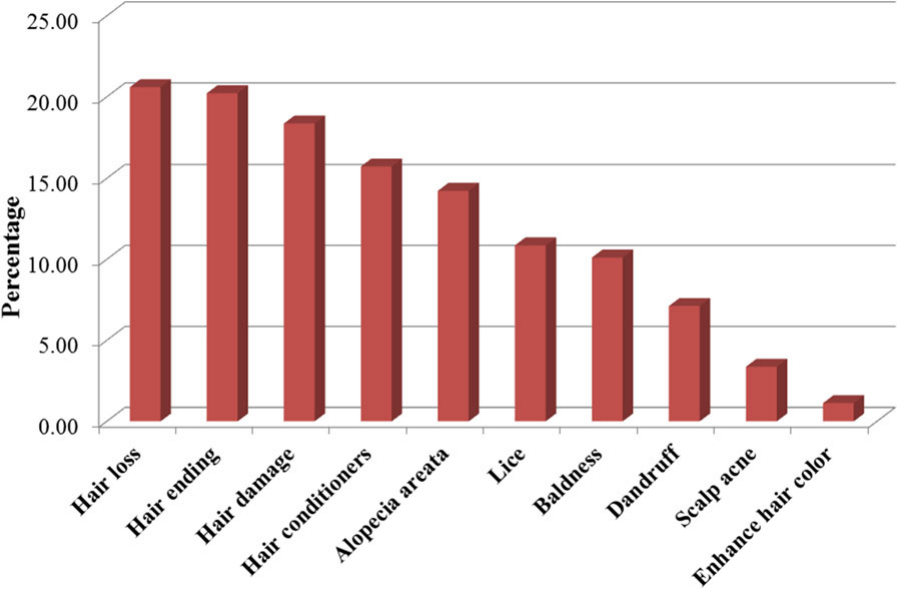
Hair and scalp disorders treated by minerals and/or animals product

As can be seen in Figs. 4 and 5, hair loss was the most common hair disorder treated with natural products including herbal, mineral or/and plant remedies. This was then followed by hair ending damage, dandruff, hair conditioners and lice whilst scabies was the least common disorder.

Finally, most of the reported natural products in this study are edible herb, mineral or animal products such as honey, eggs and milk derivatives. Moreover, they are used externally and so minor restrictions would be applied, as they have the minimum risk in comparison to the synthetic cosmetic or cosmeceutical products. This excludes the unsuitable preparation methods that were mentioned before.

This study is of great importance as it helps to preserve and improve the knowledge of herbal, mineral and animal products used as cosmeceuticals and nutraceuticals, for hair and scalp disorders, by women in the West Bank.

In the present study *Olea europaea*, *Lawsonia inermis, Ricinus communis, Allium sativum, Aloe vera, Cocos nucifera, Trigonella arabica*, *Prunus dulcis*, *Citrus limon*, *Pimpinella anisum, Punica granatum* and *Sesamum indicum* were the most frequently used plants as home remedies for the treatments of the hair and scalp. Table 9 summarizes published cosmeceutical and skin uses of these frequently used plants.

## Conclusion

Many, different, plant species are still, currently being used by herbalists and traditional practitioner healers in Palestine today, for the treatment of various types of medical conditions. This is the first study that assesses the usage, by Palestinian women, of these natural products as cosmetics or cosmeceuticals for hair and scalp disorders and afflictions. Moreover, this study is of great importance as it improves our understanding of the folkloric use of these natural products. A combined scientific effort between informants and the scientific community, working in this field, may help in the discovery of new cosmetics, cosmeceutical and nutraceutical products. Moreover, pharmacists should play a much more significant role in the preparation of suitable formulations of these products, in order to improve user compliance toward these products.

## Abbreviations

F_ic_: Informant’s Consensus
Fl: Fidelity Level
IRB: Institutional Review Board
SPSS: Statistical Package for Social Sciences
UV: Use-Value
WHO: World Health Organization
